# ﻿Four new species of *Anyphaena* Sundevall, 1833 from Xizang, China (Araneae, Anyphaenidae)

**DOI:** 10.3897/zookeys.1196.119509

**Published:** 2024-03-21

**Authors:** Shikai Li, Shilin Wang, Xiaoqi Mi, Cheng Wang

**Affiliations:** 1 Guizhou Provincial Key Laboratory for Biodiversity Conservation and Utilization in the Fanjing Mountain Region, Tongren University, Tongren, 554300 Guizhou, China Tongren University Tongren China

**Keywords:** DNA barcodes, ghost spider, morphology, southwest China, taxonomy

## Abstract

Four new species of the genus *Anyphaena* Sundevall, 1833 collected from Xizang, China, are described: *A.cibagou* Wang & Mi, **sp. nov.** (♂♀), *A.linzhi* Wang & Mi, **sp. nov.** (♂♀), *A.shufui* Wang & Mi, **sp. nov.** (♀) and *A.yejiei* Wang & Mi, **sp. nov.** (♀). Diagnostic photos of the habitus and copulatory organs and a distributional map are provided.

## ﻿Introduction

*Anyphaena* Sundevall, 1833, the most species-rich genus of the family Anyphaenidae Bertkau,1878, is represented by 106 wander-hunting species widely distributed in Asia, Europe and the Americas (WSC 2024; Rivera-Quiroz and Álvarez-Padilla 2023). In contrast to the taxonomic study of the genus in the Americas, it remains poorly known in Asia, which only has 17 species records, most of them sporadically described and only known from the original description (Durán-Barrón et al. 2016; WSC 2024). To date, eight endemic species known from both sexes are recorded from China, which is much higher than in nearby countries, such as Japan (3), Russia (3) and India (1) (WSC 2024). Among the Chinese species, half of them were described by Lin et al. (2021), including the only species recorded in Xizang, China.

Recently, spider surveys in two National Nature Reserves in Linzhi City, Xizang, China, were carried out, and more than twenty specimens of Anyphaenidae have been collected. After examination, four species belonging to *Anyphaena* are recognized as new to science and described herein.

## ﻿Material and methods

Specimens were collected by beating shrubs or hand collecting. They were preserved in 90% ethanol. Specimens are deposited in the museum of Tongren University (TRU) in Tongren, China. They were examined with an Olympus SZX 16 stereomicroscope. After dissection, the vulvae were cleared in trypsin enzyme solution before examination and imaging. Left male palps were used for the descriptions and illustrations. Photographs of the copulatory organs and habitus were taken with a Kuy Nice CCD camera mounted on an Olympus BX43 compound microscope. Compound focus images were generated using Helicon Focus v. 6.7.1. Drawings of the schematic course of the copulatory duct were generated by Adobe Illustrator CC 2018. ArcGIS v. 10.4 software was used to create a distribution map.

A partial fragment of the mitochondrial cytochrome oxidase subunit I (COI) gene of the four species was amplified and sequenced using the primers LCOI1490 and HCOI2198 (Folmer et al. 1994). The accession numbers are provided in Table 1. The pairwise genetic distances (Kimura two-parameter [K2P]) (see Table 2) were calculated using MEGA 6.0 to assess the genetic differences (Li and Zhang 2023).

**Table 1.** Voucher specimen information.

Species	Voucher code	Sex	GenBank accession number
*Anyphaenacibagou* Wang & Mi, sp. nov.	TRU-XZ-ANY-0001	♂	PP356956
	TRU-XZ-ANY-0002	♀	PP356957
*A.linzhi* Wang & Mi, sp. nov.	TRU-XZ-ANY-0005	♂	PP356958
	TRU-XZ-ANY-0006	♀	PP356962
*A.shufui* Wang & Mi, sp. nov.	TRU-XZ-ANY-0014	♀	PP356960
	TRU-XZ-ANY-0015	♀	PP356961
*A.yejiei* Wang & Mi, sp. nov.	TRU-XZ-ANY-0017	♀	PP356959
	TRU-XZ-ANY-0018	♀	PP356963
	TRU-XZ-ANY-0019	♀	PP356964

**Table 2.** Intraspecific and interspecific nucleotide divergences for four *Anyphaena* species using Kimura two-parameter model.

Species	ANY-0001	ANY-0002	ANY-0005	ANY-0006	ANY-0017	ANY-0018	ANY-0019	ANY-0014	ANY-0015
*A.cibagou* ANY-0001									
*A.cibagou* ANY-0002	0.000								
*A.linzhi* ANY-0005	0.019	0.019							
*A.linzhi* ANY-0006	0.016	0.016	0.003						
*A.yejiei* ANY-0017	0.019	0.019	0.022	0.019					
*A.yejiei* ANY-0018	0.019	0.019	0.022	0.019	0.000				
*A.yejiei* ANY-0019	0.019	0.019	0.022	0.019	0.000	0.000			
*A.shufui* ANY-0014	0.033	0.033	0.034	0.033	0.036	0.036	0.036		
*A.shufui* ANY-0015	0.033	0.033	0.034	0.033	0.036	0.036	0.036	0.000	

All measurements are given in millimetres. Leg measurements are given as total length (femur, patella, tibia, metatarsus, tarsus). References to figures in the cited papers are listed in lowercase type (fig. or figs), and figures in this paper are noted with an initial capital (Fig. or Figs). Abbreviations used in the text and figures are as follows:

**AG** accessory gland; **ALE** anterior lateral eye; **AME** anterior median eye; **At** atrium; **C** conductor; **CD** copulatory duct; **E** embolus; **FD** fertilization duct; **MA** median apophysis; **MS** median septum; **PLE** posterior lateral eye; **PME** posterior median eye; **PME** prolateral patellar apophysis; **PTA** prolateral tibia apophysis; **RTA** retrolateral tibia apophysis; **VTA** ventral tibial apophysis; **S** spermatheca.

## ﻿Taxonomy

### ﻿Family Anyphaenidae Bertkau,1878

**Genus *Anyphaena* Sundevall, 1833**

#### ﻿ Anyphaena cibagou

Taxon classificationAnimaliaAraneaeAnyphaenidae

Wang & Mi sp. nov.

45EDDA98-FE59-545D-8AB8-3D2B6B4683AA

https://zoobank.org/1179C7E2-BBB7-42DB-94D2-9A7857BA90A7

**Figure 1.**
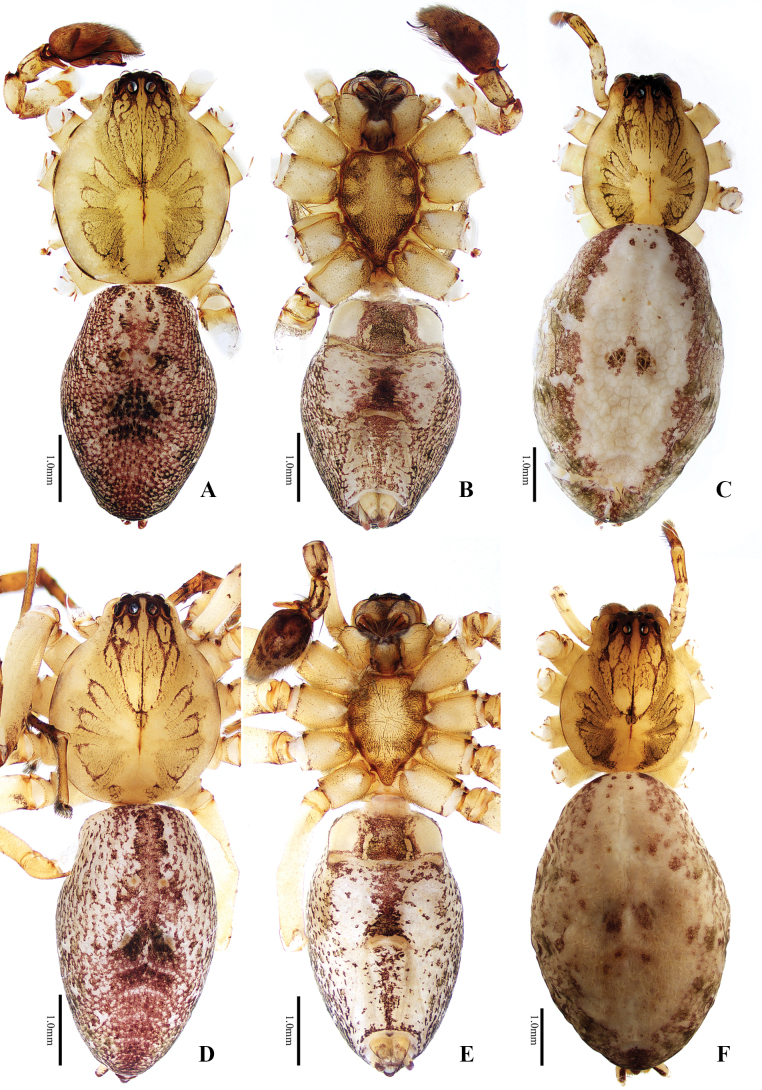
Habitus of *Anyphaena* spp. **A–C***A.cibagou* Wang & Mi, sp. nov. **D–F***A.linzhi* Wang & Mi, sp. nov. **A, B, D, E** male holotypes and **C, F** female paratypes **A, C, D, F** dorsal view **B, E** ventral view. Scale bars: 1.0 mm.

**Figure 2.**
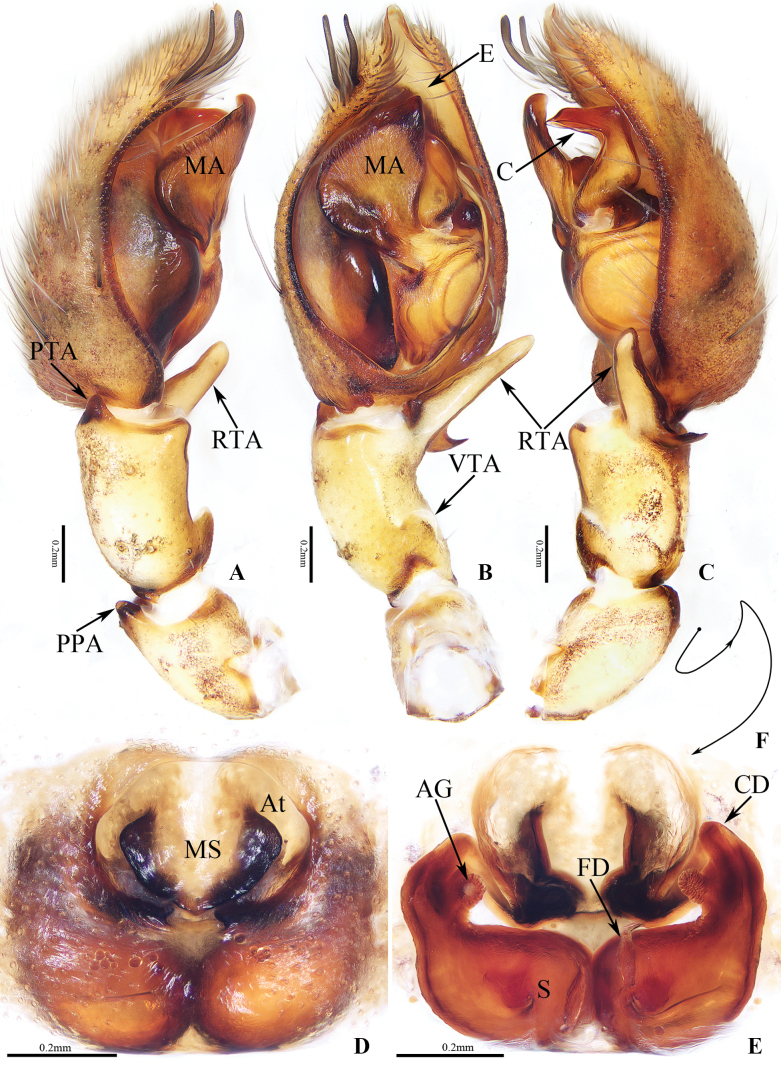
Copulatory organs of *Anyphaenacibagou* Wang & Mi, sp. nov., male holotype and female paratype **A** male palp, prolateral view **B** ditto, ventral view **C** ditto, retrolateral view **D** epigyne, ventral view **E** vulva, dorsal view **F** schematic course of copulatory duct, dorsal view. Scale bars: 0.2 mm.

Figs 1A–C , 2 , 4A , 6A

##### Type material.

***Holotype*** ♂ (TRU-XZ-ANY-0001), China: Xizang Autonomous Region, Linzhi City, Chayu County, Cibagou National Nature Reserve (28°36.03′N, 97°4.01′E, ca 2200 m), 14 Aug. 2023, C. Wang and H. Yao leg. ***Paratypes*** 1♀ (TRU-XZ-ANY-0002), same data as for holotype; 1♀ (TRU-XZ-ANY-0003), Cibagou National Nature Reserve (28°41.43′N, 97°2.86′E, ca 2570 m), 25 Jun. 2023, C. Wang leg.

##### Etymology.

The specific name is a noun in apposition and refers to the type of locality, Cibagou National Nature Reserve.

##### Diagnosis.

**Figure 3:**
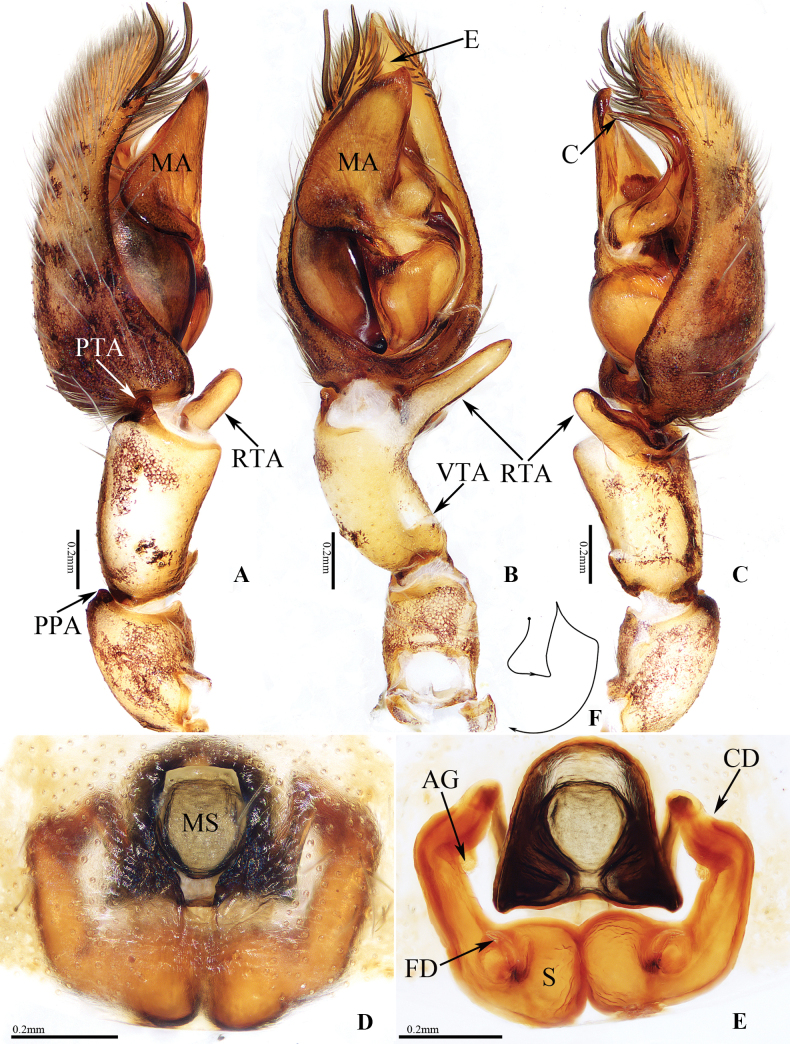
. Copulatory organs of *Anyphaenalinzhi* Wang & Mi, sp. nov., male holotype and female paratype **A** male palp, prolateral view **B** ditto, ventral view **C** ditto, retrolateral view **D** epigyne, ventral view **E** vulva, dorsal view **F** schematic course of copulatory duct, dorsal view. Scale bars: 0.2 mm.

**Figure 4.**
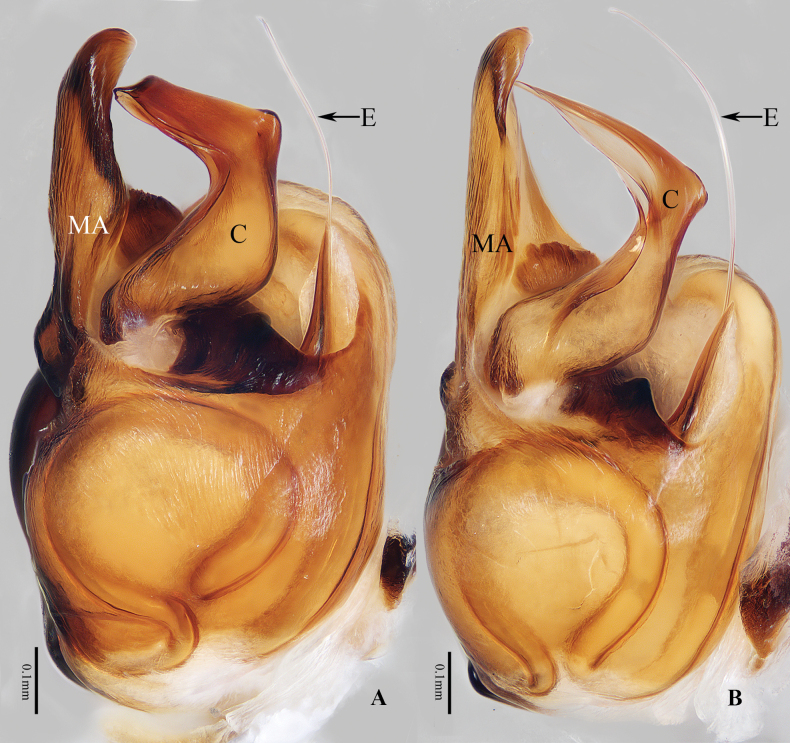
Bulb of *Anyphaena* spp., retrolateral view **A***A.cibagou* Wang & Mi, sp. nov., holotype **B***A.linzhi* Wang & Mi, sp. nov., paratype. Scale bars: 0.1 mm.

The species is closely similar to that of *A.linzhi* sp. nov., in habitus and copulatory organs, but it can be easily distinguished by the following: 1) the main portion of the median apophysis is almost oval, and slightly longer than wide in ventral view (Fig. 2B), vs elongate-oval, more than two times longer than wide in *A.linzhi* sp. nov. (Fig. 3B); 2) the conductor is acutely narrowed distally (Fig. 4A), vs almost tapered at distal half in *A.linzhi* sp. nov. (Fig. 4B); 3) the atrium is wider than long, and the median septum has a pair of laterally extended lamellar processes (Fig. 2D), vs atrium is longer than wide, and the median septum lacks similar processes in *A.linzhi* sp. nov. (Fig. 3D); 4) the accessory glands are located terminally on copulatory ducts (Fig. 2E), vs located medially in *A.linzhi* sp. nov. (Fig. 3E); and 5) the spermathecae are elongate-oval (Fig. 2E), vs almost spherical in *A.linzhi* sp. nov. (Fig. 3E). The male also somewhat resembles that of *A.tibet* Lin & Li, 2021 in having similar palp, especially the invert L-shaped conductor in retrolateral view, but it can be easily distinguished by the retrolateral tibial apophysis, which has a dorsal ramus about one-fifth the ventral ramus length and with a blunt end in retrolateral view (Fig. 2C), vs the dorsal ramus more than half the ventral ramus length and with a somewhat pointed tip in *A.tibet* (Lin et al. 2021: fig. 7C), and by the smooth conductor (Figs 2C, 4A), vs serrated on the inner margin in *A.tibet* (Lin et al. 2021: fig. 13B).

##### Description.

**Male** (holotype; Figs 1A, B, 2A–C, 4A). Total length 6.72. Carapace 3.20 long, 2.78 wide. Abdomen 3.53 long, 2.35 wide. Clypeus 0.15 high. Eye sizes: AME 0.12, ALE 0.15, PME 0.17, PLE 0.18. Legs: I 9.84 (2.24, 0.79, 3.06, 2.49, 1.26), II 8.48 (2.39, 0.75, 2.66, 1.90, 0.78), III 5.99 (1.95, 0.65, 1.62, 1.26, 0.51), IV 8.67 (2.46, 0.64, 2.34, 2.41, 0.82). Carapace pale yellow to brown, with sub-oval thorax, slightly elevated cephalon, and big, irregular, brown markings; fovea longitudinal, dark red. Chelicerae red-brown, with four promarginal and seven retromarginal teeth. Endites yellow, longer than wide, bearing dense dark setae on inner portion of anterior margins. Labium darker than endites, bearing dark setae at distal margin. Sternum yellow to red-brown, covered with dense short setae, and with three pairs of anteromedian yellow spots laterally. Legs yellow to red-brown, with triangular ventral apophyses on base of coxae. Abdomen elongate-oval, dorsum red-brown to dark brown, spotted, with longitudinal, anterior pale band, irregular, median, dark patch, and two pairs of medium muscle depressions; venter pale to red-brown.

Palp (Figs 2A–C, 4A): patella slightly longer than wide, with short, sclerotized distro-prolateral apophysis less than one-tenth its length; tibia about 1.5 times longer than wide, with half-round, base-ventral apophysis and bifurcated retrolateral apophysis, which has long, bar-shaped ventral ramus directed towards ca 11:30 o’clock position apically in retrolateral view, and short, lamellar dorsal ramus; cymbium setose, bearing two distro-prolateral macro-setose; bulb almost oval; tegulum swollen; subtegulum elongated, prolaterally located; median apophysis originates from the medium of retrolateral side of bulb, the main portion almost oval, and with rather pointed tip slightly curved dorsally; embolus thin, weakly sclerotized, partly hidden by conductor in ventral view; conductor anterior to the base of median apophysis, curved into invert L-shape in retrolateral view.

**Female** (TRU-XZ-ANY-0002; Figs 1C, 2D–F). Total length 8.32. Carapace 3.05 long, 2.36 wide. Abdomen 5.59 long, 3.54 wide. Clypeus 0.17 high. Eye sizes: AME 0.11, ALE 0.15, PME 0.16, PLE 0.17. Measurements of legs: I 11.15 (3.03, 1.09, 3.20, 2.48, 1.35), II 9.78 (2.75, 0.93, 2.76, 2.17, 1.17), III 7.56 (2.28, 0.88, 1.74, 1.79, 0.87), IV 10.49 (3.04, 0.92, 2.51, 2.92, 1.10). Habitus (Fig. 1C) similar to that of male except having broad, longitudinal, pale band extending across the whole surface, only with sex retromarginal cheliceral teeth and lacking ventral apophysis on the base of coaxe.

Epigyne-vulva (Fig. 2D–F): wider than long, with anteriorly located, oval atrium more than half the epigynal width; median septum medially located on atrium, with strongly sclerotized, laterally extended lamellar processes; copulatory openings invisible; copulatory ducts strongly curved at base and then gradually thickened to connected to the anterolateral portions of elongate-oval, touched spermathecae, with short, terminal accessory glands; fertilization ducts lamellar, originate from the inner-base of spermathecae.

##### Distribution.

Known only from the type locality in Xizang, China (Fig. 6A).

#### ﻿ Anyphaena linzhi

Taxon classificationAnimaliaAraneaeAnyphaenidae

Wang & Mi sp. nov.

7A21F229-13ED-5C1D-9A79-42B3089BC7BB

https://zoobank.org/2D1BE0AA-F5DF-48BB-8B5F-79042CA311BC

Figs 1D–F , 3 , 4B , 6A

##### Type material.

***Holotype*** ♂ (TRU-XZ-ANY-0004), China: Xizang Autonomous Region, Linzhi City, Bomi County, Gangyunshanlin Scenic Area (29°52.99′N, 95°33.59′E, ca 2680 m), 29 Jun. 2023, C. Wang leg. ***Paratypes*** 1♂7♀ (TRU-XZ-ANY-0005–0012), same data as for holotype.

##### Etymology.

The species name is a noun in apposition and comes from the type locality, Linzhi City.

##### Diagnosis.

*Anyphaenalinzhi* sp. nov. closely resembles that of *A.cibagou* sp. nov., but it can be distinguished by the following: 1) the main portion of median apophysis is elongate-oval, more than two times longer than wide in ventral view (Fig. 3B), vs almost oval, and slightly longer than wide in *A.cibagou* sp. nov. (Fig. 2B); 2) the conductor is tapered at distal half in retrolateral view (Fig. 4B), vs acutely narrowed distally in *A.cibagou* sp. nov. (Fig. 4A); 3) the atrium is longer than wide, and the median septum lacks process (Fig. 3D), vs atrium is wider than long, and the median septum has laterally extended lamellar processes in *A.cibagou* sp. nov. (Fig. 2D); 4) the accessory glands are located medially on copulatory ducts (Fig. 3E), vs located terminally in *A.cibagou* sp. nov. (Fig. 2E); and 5) the spermathecae are about spherical (Fig. 3E), vs elongate-oval in *A.cibagou* sp. nov. (Fig. 2E). The male also somewhat resembles that of *A.tibet* Lin & Li, 2021 in having very similar palpal structure, but it differs in: 1) the ventral ramus of retrolateral tibial apophysis is anteroventrally extending, and about three times longer than the dorsal ramus in retrolateral view (Fig. 3C), vs upward extending, and less than two times longer than the dorsal ramus in *A.tibet* (Lin et al. 2021: fig. 7C); 2) the conductor is smooth (Figs 3C, 4B), vs serrated on the inner margin in *A.tibet* (Lin et al. 2021: fig. 13B).

##### Description.

**Male** (holotype; Figs 1D, E, 3A–C, 4B). Total length 6.52. Carapace 2.82 long, 2.36 wide. Abdomen 3.76 long, 2.25 wide. Clypeus 0.11 high. Eye sizes: AME 0.12, ALE 0.16, PME 0.15, PLE 0.16. Measurements of legs: I 13.27 (3.42, 1.10, 4.12, 3.09, 1.54), II 11.87 (3.14, 1.06, 3.68, 2.69, 1.30), III 8.54 (2.66, 0.79, 2.24, 2.06, 0.79), IV 12.02 (3.31, 0.94, 3.17, 3.46, 1.14). Carapace almost oval, with elevated cephalon, and big, irregular, brown markings; fovea longitudinal, red-brown. Chelicerae yellow to gray-brown, with four promarginal and eight retromarginal teeth. Endites longer than wide, bearing clusters of dark-brown setae on inner portion of anterior margins. Labium darker than endites. Sternum almost heart-shaped, setose. Legs yellow-brown, with sub-triangular apophyses on the base of coxae. Abdomen elongated, dorsum pale to red-brown, with longitudinal, anteromedian pale band followed by two pairs of muscle depressions and two irregular dark patches medially; venter paler to dark brown.

Palp (Figs 3A–C, 4B): patella slightly longer than wide, with sclerotized, disto-prolateral apophysis; tibia slightly curved medially, with almost half-round ventro-retrolateral apophysis at base and bifurcated disto-retrolateral apophysis, which has straight, bar-shaped ventral ramus directed towards ca 10 o’clock position apically in retrolateral view, and strongly sclerotized, lamellar dorsal ramus; cymbium longer than wide, with two slender, medially curved macrosetea on the distal portion of prolateral margin; bulb almost oval; tegulum swollen; subtegulum elongated, prolaterally located; median apophysis originates from the middle of retrolateral side of bulb, main portion elongated, with somewhat pointed tip; embolus thin, partly visible; conductor retrolateral to the main portion of median apophysis, strongly curved medially, and with tapered distal half extending anteroventrally.

**Female** (TRU-XZ-ANY-0006; Figs 1F, 3D–F). Total length 8.41. Carapace 3.05 long, 2.50 wide. Abdomen 5.50 long, 3.64 wide. Clypeus 0.12 high. Eye sizes AME 0.10, ALE 0.15, PME 0.14, PLE 0.15. Measurements of legs: I 12.89 (3.26, 1.39, 3.60, 3.06, 1.58), II 11.40 (3.00, 1.22, 3.18, 2.74, 1.26), III 8.85 (2.52, 1.16, 2.05, 2.18, 0.94), IV 11.73 (3.12, 1.20, 2.87, 3.38, 1.16). Habitus (Fig. 1F) similar to that of male except having the dorsal abdominal pale band extending the whole surface, seven retromarginal cheliceral teeth, and lacking ventral apophysis on the base of coaxe.

Epigyne and vulva (Fig. 3D–F): wider than long; atrium almost hexagonal, anteriorly located; median septum almost linguiform; copulatory ducts curved, gradually thicken, with short, medially located accessory glands less than one-third the largest diameter of copulatory ducts in length; spermathecae sub-spherical, touched; fertilization ducts lamellar.

##### Distribution.

Known only from the type locality in Xizang, China (Fig. 6A).

#### ﻿ Anyphaena shufui

Taxon classificationAnimaliaAraneaeAnyphaenidae

Wang & Mi sp. nov.

C6475D3F-9B7A-549E-98CF-974ABC51D9D1

https://zoobank.org/F0B57275-CDBF-4315-A047-4AFB9D08EDF1

**Figure 5.**
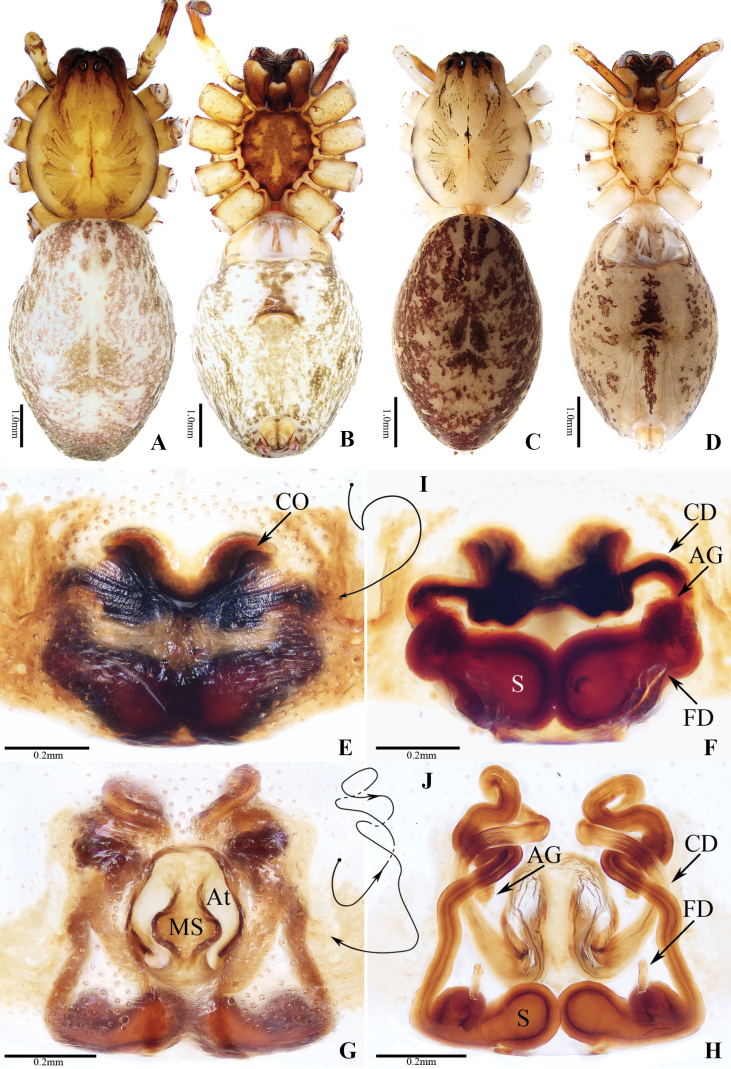
Female holotypes of *Anyphaena* spp. **A, B, E, F, I***A.shufui* Wang & Mi, sp. nov. **C, D, G, H, J***A.yejiei* Wang & Mi, sp. nov. **A, C** habitus, dorsal view **B, D** ditto, ventral view **E, G** epigyne, ventral view **F, H** vulva, dorsal view **I, J** schematic course of copulatory duct, dorsal view. Scale bars: 1.0 mm (**A–D**); 0.2 mm (**E–H**).

**Figure 6.**
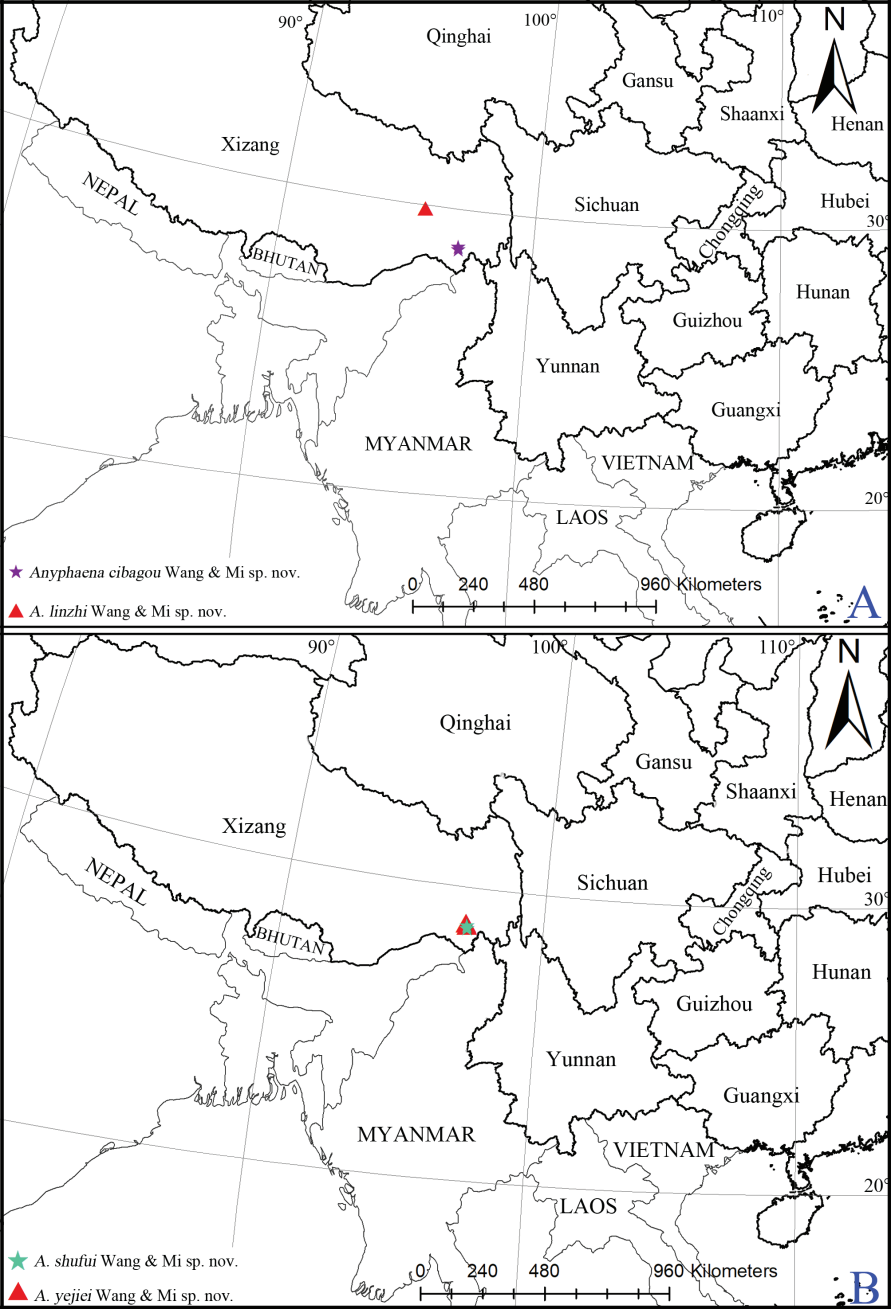
Distributional records of the *Anyphaena* spp. **A***A.cibagou* Wang & Mi, sp. nov. and *A.linzhi* Wang & Mi, sp. nov. **B***A.shufui* Wang & Mi, sp. nov. and *A.yejiei* Wang & Mi, sp. nov.

Figs 5A, B, E, F, I , 6B

##### Type material.

***Holotype*** ♀ (TRU-XZ-ANY-00013), CHINA: Xizang Autonomous Region, Linzhi City, Chayu County, Cibagou National Nature Reserve (28°36.03′N, 97°4.01′E, ca 2200 m), 14 Aug. 2023, C. Wang et al. leg. ***Paratypes*** 1♀ (TRU-XZ-ANY-0014), same data as for holotype; 1♀ (TRU-XZ-ANY-0015), Cibagou National Nature Reserve (28°41.43′N, 97°2.86′E, ca 2570 m), 25 Jun. 2023, C. Wang leg.

##### Etymology.

The species is named after Mr Fu Shu, who helped us with specimens collecting in Linzhi, Xizang; noun (name) in genitive case.

##### Diagnosis.

*Anyphaenashufui* sp. nov. closely resembles that of *A.rhynchophysa* Feng, Ma & Yang, 2012 in epigyne-vulva structure, but it can be easily distinguished by the atrium, which is slit-shaped (Fig. 5E), vs oval in *A.rhynchophysa* (Feng et al. 2012: fig. 8).

##### Description.

**Female** (holotype; Fig. 5 A, B, E, F, I). Total length 8.95. Carapace 3.95 long, 3.19 wide. Abdomen 5.76 long, 3.90 wide. Clypeus 0.30 high. Eye sizes: AME 0.11, ALE 0.20, PME 0.19, PLE 0.20. Legs: I 14.99 (4.14, 1.22, 4.45, 3.40, 1.78); II 13.69 (3.91, 1.21, 3.83, 3.25, 1.49); III 10.61 (3.23, 1.08, 2.65, 2.53, 1.12); IV 14.71 (4.40, 1.24, 3.64, 4.12, 1.31). Carapace yellow to brown, with oval thorax and elevated cephalon, bearing big, irregular brown markings; fovea longitudinal, dark red. Chelicerae red-brown, with three promarginal and eight retromarginal teeth. Endites red-brown, ca two times longer than wide. Labium colored as endites. Sternum red-brown, setose, with irregular dark yellow stripes. Legs yellow to brown. Abdomen elongated, dorsum pale to brown, with anterior, longitudinal irregular pale band followed by brown markings, and two pairs of muscle depressions; venter paler than dorsum.

Epigyne-vulva (Fig. 5E, F, I): wider than long; atrium anteriorly located, slit-shaped; copulatory openings located on the lateral sides of atrium; copulatory ducts widened at base, and then folded and acutely narrowed to tube-shaped portions, which curved medially and with oval, terminal accessory glands; spermathecae elongate-oval, touched; fertilization ducts lamellar.

**Male.** Unknown.

##### Distribution.

Known only from the type locality in Xizang, China (Fig. 6B).

#### ﻿ Anyphaena yejiei

Taxon classificationAnimaliaAraneaeAnyphaenidae

Wang & Mi sp. nov.

A8CA2D01-793D-5D0E-939C-168E5EB62416

https://zoobank.org/71FE6C75-DCE8-4CDB-A4C6-8136566069C7

Figs 5C, D, G, H, J , 6B

##### Type material.

***Holotype*** ♀ (TRU-XZ-ANY-00016), China: Xizang Autonomous Region, Linzhi City, Chayu County, Cibagou National Nature Reserve (28°46.62′N, 97°0.86′E, ca 2880 m), 24 Jun. 2023, C. Wang et al. leg. ***Paratypes*** 4♀ (TRU-XZ-ANY-0017–0020), same data as for holotype; 2♀ (TRU-XZ-ANY-0021–0022), Cibagou National Nature Reserve (28°41.43′N, 97°2.86′E, ca 2570 m), 25 Jun. 2023, C. Wang leg.

##### Etymology.

The species is named after Mr Yejie Lin, who contributed to the taxonomic study of Chinese *Anyphaena* species and helped with species identification; noun (name) in genitive case.

##### Diagnosis.

*Anyphaenayejiei* sp. nov. is similar to that of *A.shenzhen* Lin & Li, 2021 in having a very long, distorted copulatory duct, but it can be easily distinguished by the medially located atrium and medially originated copulatory duct (Fig. 5G, H), vs anteriorly located atrium and anteriorly originated copulatory duct in *A.shenzhen* (Lin et al. 2021: fig. 6A, B). It also resembles that of *A.cibagou* sp. nov. in having a similar median septum, but it can be easily distinguished by the medially located atrium and much thinner and coiled copulatory ducts (Fig. 5G, H), vs anteriorly located atrium and much thicken, and not coiled copulatory ducts in *A.cibagou* sp. nov. (Fig. 2D, E).

##### Description.

**Female** (Fig. 5C, D, G, H, J). Total length 7.84. Carapace 3.00 long, 2.44 wide. Abdomen 4.72 long, 3.00 wide. Clypeus height 0.17. Eye sizes: AME 0.11, ALE 0.16, PME 0.15, PLE 0.17. Measurements of legs: I 7.92 (2.12, 0.75, 2.10, 1.94, 1.01); II 7.18 (1.95, 0.78, 1.77, 1.72, 0.96); III 5.15 (1.36, 0.60, 1.14, 1.46, 0.59); IV 7.39 (2.05, 0.70, 1.77, 2.23, 0.64). Carapace pale yellow to brown, with sub-oval thorax and elevated cephalon bearing big, brown markings; fovea longitudinal, dark red. Chelicerae red-brown, with four promarginal and seven retromarginal teeth. Endites dark yellow, almost paralleled. Labium dark brown, with pale distal portion bearing dense dark setae. Sternum yellow, almost heart-shaped, with small dark-brown spots. Legs pale to brown. Abdomen elongated, dorsum fuchsia, with irregular yellow and fuchsia markings; venter pale, covered with brown spots laterally.

Epigyne-vulva (Fig. 5G, H, J): longer than wide, with oval, medially located atrium separated by the sub-oval septum; copulatory openings beneath the lateral margin of atrium; copulatory ducts long, forming complicated coils and with medially located, bar-shaped accessory glands extending downward; spermathecae elongated, touched, with two sub-spherical portions; fertilization ducts lamellar, originate at the anterior portions of the outside spherical potions of spermathecae.

**Male.** Unknown.

##### Distribution.

Known only from the type locality in Xizang, China (Fig. 6B).

## Supplementary Material

XML Treatment for Anyphaena cibagou

XML Treatment for Anyphaena linzhi

XML Treatment for Anyphaena shufui

XML Treatment for Anyphaena yejiei

## ﻿Additional information

### Author ORCIDs

Shikai Li https://orcid.org/0009-0002-3947-2550

Shilin Wang https://orcid.org/0009-0003-0759-5350

Xiaoqi Mi https://orcid.org/0000-0003-1744-3855

Cheng Wang https://orcid.org/0000-0003-1831-0579

### Data availability

All of the data that support the findings of this study are available in the main text.
